# Prompt Hematological Recovery in Response to a Combination of Pegylated Interferon α-2a and Rituximab in a Profoundly Immuno-Suppressed Hairy Cell Leukemia Patient with a Mycobacterial Infection at Onset: Benefits and Drawbacks of Rapid Immune Reconstitution

**DOI:** 10.3390/hematolrep14020020

**Published:** 2022-04-13

**Authors:** Anna Furlan, Maria Cristina Rossi, Filippo Gherlinzoni, Piergiorgio Scotton

**Affiliations:** 1 Hematology Unit, Azienda ULSS2 Marca Trevigiana, 31100 Treviso, Italy; filippo.gherlinzoni@aulss2.veneto.it; 2 Infectious Disease Unit, Azienda ULSS2 Marca Trevigiana, 31100 Treviso, Italy; mariacristina.rossi@aulss2.veneto.it (M.C.R.); piergiorgio.scotton@aulss2.veneto.it (P.S.)

**Keywords:** hairy cell leukemia, interferon, rituximab, opportunistic infections, immune reconstitution inflammatory syndrome

## Abstract

The present paper reports, to the best of our knowledge for the first time, the efficacy and tolerability of the combination of interferon (IFN)α-2a in pegylated formulation and rituximab after a “priming” phase with IFN in the frontline treatment of hairy cell leukemia (HCL) in a profoundly immunosuppressed patient with a *Mycobacterium abscessus* infection at onset. This immunotherapy combination may represent a potential therapeutic option in patients with active severe infection and for whom the use of purine nucleoside analogues (PNA) is contraindicated. The benefits and drawbacks of remarkably rapid immune reconstitution in the context of opportunistic infections are highlighted as well, as the potentially paradoxical effects of immune recovery as a result of effective immunotherapy strategies, known as immune reconstitution inflammatory syndrome (IRIS), have to be taken into account when dealing with patients with opportunistic infections.

## 1. Introduction

Classical hairy cell leukemia (HCL) is a B-cell chronic lymphoproliferative disorder characterized by splenomegaly, pancytopenia, and bone marrow involvement with fibrosis. Patients with HCL should be treated if they have symptoms from the disease, progressive cytopenias, or splenomegaly. Chemotherapy with purine nucleoside analogues (PNA), generally either cladribine or pentostatin in monotherapy, remains the standard first-line treatment of HCL for physically fit and symptomatic HCL patients, in most cases conferring long overall survival (OS). Chemoimmunotherapy with cladribine followed by the anti-CD20 monoclonal antibody rituximab, as well as adding concurrent rituximab to cladribine in first-line therapy, have been demonstrated to improve the rate of durable CR [1,2]. One of the most challenging clinical situations involves patients with symptomatic HCL and febrile infection. If it is not possible to control the infection prior to instituting treatment with PNA, the use of alpha-interferon (IFN-α) maybe transiently required in order to increase the neutrophil count prior to beginning PNA [3].

Immune recovery in the context of opportunistic infections such as mycobacteriosis may be associated with paradoxical effects secondary to a dysregulated and hyperinflammatory response known as immune reconstitution inflammatory syndrome (IRIS). Opportunistic infections can initially be latent or have subacute presentations, only become clinically apparent and/or invasive or disseminated after recovery of immunocompetence. While IRIS typically occurs in the first months of treatment in HIV/AIDS patients, it has been described in non-HIV related conditions as well, including profound neutropenia [4].

This paper reports the efficacy and tolerability of a combination of IFNα-2a in its pegylated formulation with rituximab following a “priming” phase with IFN for the frontline treatment of hairy cell leukemia (HCL) in a profoundly immunosuppressed patient with a *Mycobacterium abscessus* infection at onset. 

The *Mycobacterium abscessus* complex comprises a group of rapidly growing, multidrug-resistant, nontuberculous mycobacteria (NTM) that are responsible for a wide spectrum of skin and soft tissue diseases, central nervous system, and ocular infections and bacteremia [5]. The containment of NTM infections may be hampered in patients with HCL due to severe impairment of both the innate and adaptive immune responses. The first implies recruitment of macrophages and neutrophils to the site of infection at an early stage. When phagosomal defence mechanisms fail, adaptive immunity has an important role in granuloma maturation through the recruitment of B and T cells. 

The paradoxical effects of a remarkably rapid immune reconstitution as a result of effective immunotherapy in the setting of an NTM infection will be highlighted in this paper.

## 2. Case Description

A 73-year-old man was hospitalized (day 0) due to persistent night fevers spiking up to 39 °C and a dry cough. His past medical history was significant for coronary artery disease, hypertension, hypercholesterolemia, and benign prostatic hyperplasia.

Laboratory examinations at hospitalization were significant for pancytopenia (WBC 1.00 × 10^3^/μL, absolute neutrophil count 0.47 × 10^3^/μL, absolute lymphocyte count 0.50 × 10^3^/μL, absolute monocyte count 0.02 × 10^3^/μL, hemoglobin 10.5 g/dL, platelets 67 × 10^3^/μL, and reticulocyte count 38 × 10^3^/μL. Flow cytometry studies on peripheral blood detected a B cell population representing 30–35% of B lymphocytes with k light chain restriction and coexpression of CD11c, CD19, CD20, CD25, and CD103, consistent with hairy cell leukemia (HCL) in the context of marked lymphocytopenia (B cells 40.1/mmc, T CD4+ 272.5/mmc, T CD8+ 131.3/mmc, CD4/CD8 ratio 2, NK 12.7/mmc). A bone marrow biopsy showed extensive interstitial and nodular infiltration of medium-sized lymphoid cells with moderately abundant cytoplasm, reniform nuclei, and open chromatin, with positive staining for CD20, Annexin A1, and Cyclin D1.

While a standard X-ray was unremarkable, a chest CT scan documented multiple hilar and mediastinal lymph nodes (the larger of which was 1.5 cm in diameter and partially calcified) and a moderate splenomegaly (13 cm in bipolar diameter).

Blood cultures drawn the day before admission and at six subsequent samplings turned out to be positive for *Mycobacterium abscessus* subsp. *massilliense*. SARS-CoV-2 RT-PCR on nasopharyngeal swab, Cytomegalovirus viremia, cryptococcal antigen, serum galactomannan and beta-D-Glucan assays, Bartonella and Strongyloides serologies, acid-fast bacilli (AFB) stain, and culture on sputum were all negative. 

On day 8 the patient was transferred to the infectious disease unit and started on empiric combination antibiotic therapy with amikacin, clofazimine, tigecyclin, azithromycin, and imipenem. Profound neutropenia and monocytopenia persisted despite an attempt at stimulation with granulocyte colony-stimulating factor (G-CSF). Therefore, it was decided to start the patient on leukemia-directed therapy in an attempt to achieve recovery of immunological competence as a requirement for clinical recovery from the infection. The purine nucleoside analogues (PNAs) currently used for first-line treatment of HCL were considered to be unsafe in the setting of a mycobacterial infection due their heavy suppressive effect on cellular immunity [6].

IFNα represents a feasible option for the treatment of HCL patients with active severe infections for whom the use of PNAs is contraindicated. Due to the unavailability of IFNα, pegylated (PEG) IFNα-2a was prescribed off-label and administered (day 13) at a starting dose of 135 mcg once weekly, based on a previous report on patients with myeloproliferative disorders [7]. Ten days later (day 24), after the of BRAF V600E mutation results were confirmed to be positive and the oral BRAF inhibitor vemurafenib was made available, PEG IFNα-2a was discontinued after the second dose and the patient was started on the association of rituximab and vemurafenib based on the results of a phase 2 trial on patients with refractory or relapsed HCL [8]. Vemurafenib was discontinued after a total of only nine twice-daily doses due to cardiotoxicity (QT interval prolongation) and a grade 3 skin reaction.

**Figure 1:**
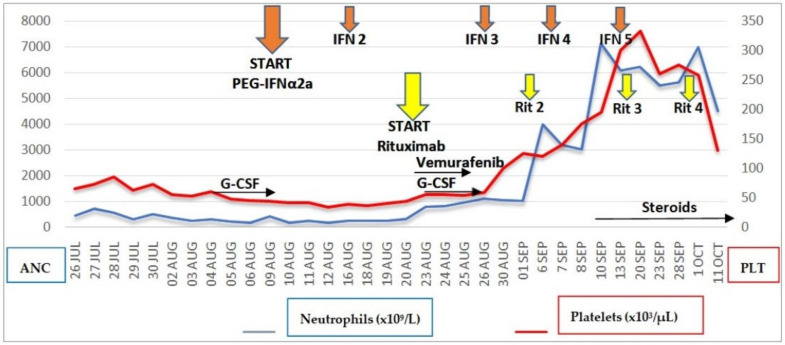
Trends of neutrophil and platelet counts in relation to the timing of PEG IFNα-2a, rituximab, vemurafenib, and G-CSF administration. The Y-axes on the left and right sides refer to ANC and PLT, respectively. Absolute neutrophil count recovery >1.00 × 10^3^/μL occurred on day +6 from the first dose of rituximab, corresponding to day +17 from the first dose of PEG IFNα-2a. G-CSF was administered from day +1 to day +7 from the first dose of rituximab. ANC, absolute neutrophil count; PLT, platelet count.

Despite early withdrawal of vemurafenib, blood counts documented absolute neutrophil count recovery >1.00 × 10^3^/μL on day +6 from the first dose of rituximab, corresponding to day +17 from starting PEG IFNα-2a. G-CSF stimulation, reintroduced the day after the first rituximab dose, was definitively stopped after confirming ANC >1.00 × 10^3^/μL on two consecutive days. The platelet count recovered to a level >100 × 10^3^/μL on day +10 from the first dose of rituximab and normalized on day +18 (Figure 1).

Vemurafenib was permanently discontinued in view of its toxicity and the prompt hematological response independent of vemurafenib itself. The patient was continued on PEG IFNα-2a (five total weekly doses) in association with rituximab (four total doses every two weeks, reflecting the schedule of association with vemurafenib).

Despite complete hematological recovery and repeatedly negative blood cultures after day 18, the whole course of hospitalization was characterized by fevers, which responded to intermediate-low dose steroids (methylprednisolone/prednisone 20 mg IV/PO QD) and relapsed after several attempts at discontinuation or dose reduction. Supportive management with IV hydration and nasogastric feeding became necessary due to significant weight loss, despite an initial recovery. 

**Figure 2:**
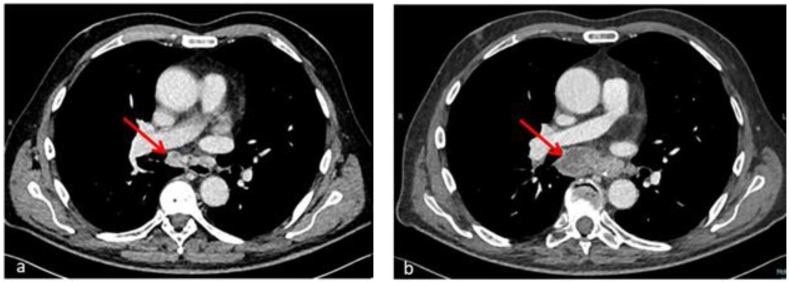
Axial chest CT sections after hematological recovery (**b**) compared with baseline examination (**a**) show enlargement of confluent hilar and mediastinal lymph nodes (66 × 26 mm vs. 24 × 15 mm) with necrosis and colliquation phenomena (red arrows) in an HCL patient with *Mycobacterium abscessus* infection. The radiological picture in the context of complete hematological remission is consistent with immune reconstitution syndrome.

A repeat chest CT scan (day 50) described enlargement of multiple confluent hilar and mediastinal lymph nodes, with overall extension of 66 × 26 mm (compared to 24 × 15 mm in the previous examination) and necrosis and colliquation phenomena (Figure 2); a new splenic hypodense subcapsular nodular lesion appeared as well, within a normal-sized spleen. Even in the absence of histologic information from lymph node biopsy, worsening of pre-existing lymphadenopathies and constitutional symptoms concomitant with immune recovery as well as steroid responsiveness were considered to be highly suggestive of IRIS.

On day 60, the patient was discharged afebrile on azithromycin, moxifloxacin, linezolid, and prednisone at 25 mg QD. Peripheral blood flow cytometry and a bone marrow biopsy performed two months after the first rituximab dose (day 75) documented complete clearance of leukemia and full T lymphocyte subset reconstitution in addition to ANC recovery.

## 3. Discussion

The clinical activity of IFNα in HCL patients has been known since the early 1980s. However, most IFN-induced responses were partial and of relatively short duration and most patients eventually relapsed after discontinuing treatment, with a median time to treatment failure ranging from 6 to 25 months [9]. PNAs such as cladribine and pentostatin have now replaced IFNα as standard first-line therapy for HCL; current NCCN guidelines suggest IFNα therapy for patients with HCL who have relapsed after treatment with PNAs, especially for those who may require maintenance therapy [10]. For patients who are pregnant or when the profound immunosuppressive side effects of PNAs must be avoided, IFNα may represent a safer initial treatment option [11].

The anti-CD20 monoclonal antibody rituximab has demonstrated significant clinical activity as a monotherapy for the treatment of relapsed or refractory indolent lymphoma [12], and is approved for use in patients with relapsed and refractory follicular lymphoma.

In patients with HCL, the efficacy of rituximab in terms of overall response (OR) and complete response (CR) rates has been demonstrated in both frontline settings and at relapse, with a reported 100% response rate when combined with PNAs even beyond frontline therapy [1,13]. However, rituximab as a monotherapy is associated with lower efficacy, with reported OR rates ranging from 25 to 80% (CR rates 10 to 55%), as well as a high relapse rate, with a median time to relapse of 17.5 months [13,14].

A small number of cases of frontline rituximab monotherapy have been recorded in the literature, with CR reported in 58% of patients [15]. Of note, rituximab retreatment has been reported to be effective [13]. Despite encouraging results, the role of rituximab monotherapy in the frontline setting needs to be determined, and it should be limited to patients who are unable to receive PNAs [10].

As suggested by preclinical findings, the administration of IFNα-2a before and during rituximab treatment could be effective in increasing CD20 antigen surface expression on neoplastic B cells [16]. Furthermore, the immunomodulatory effects of IFNα-2a, including stimulation of T cell cytotoxicity and NK cell activity [17,18], might synergize with rituximab to induce neoplastic clone suppression, mainly by enhancing antibody-dependent cell mediated cytotoxicity.

The clinical activity and safety of a combined immunotherapy approach using IFNα-2a and rituximab has been demonstrated in patients with relapsed low-grade non-Hodgkin’s lymphoma [19]. In this study, four weekly doses of rituximab were administered in association with IFNα-2a after a two-week priming phase with IFN. Notably, hematologic toxicity was mostly related to IFN and accounted for almost half of grade 3 adverse events; approximately 35% of patients required a dose reduction or a temporary discontinuation of IFN. 

The potential of IFN to enhance the effects of rituximab has been shown by a randomized phase II study in patients with previously untreated or first relapse CD20-positive low-grade lymphoma [20]. After a first cycle of four weekly rituximab doses, patients receiving rituximab plus IFN after a two-week priming phase had higher rates of OR and CR compared with patients receiving rituximab alone. However, none of the above-mentioned studies included patients with HCL.

The case reports recorded in the literature involving frontline rituximab monotherapy report follow-up achievement of CR in a time range from 1 to 54 months [15]. The prompt attainment of CR in the case described here highlights the potential role of IFNα-2a as a trigger of rituximab-induced cytotoxicity through synergistic effects. Because of very early discontinuation, vemurafenib is unlikely to have contributed significantly to the hematological response.

Prospective and retrospective studies of rituximab monotherapy both in the frontline settings and in relapsed/refractory HCL did not report concomitant use of granulocyte colony-stimulating factor (G-CSF) or granulocyte–macrophage colony-stimulating factor (GM-CSF) [13,21]. On the other hand, the administration of G-CSF in association with IFN-α in patients with HCL has been reported as a useful tool for managing or preventing neutropenic complications in the early phase of treatment [22]. In addition, a few studies have examined the effect of G-CSF [23], GM-CSF [24], and Pegfilgrastim [25] in reducing PNA treatment toxicity in patients with HCL. 

Against this background, a short G-CSF course was administered to our patient starting from day +1 after the first rituximab dose under the assumption of an ongoing antineoplastic effect of previous IFN and rituximab therapy in order to favor ANC recovery as a requirement for faster clinical recovery from infection.

In the case presented here, with the exception of a grade 2 infusion reaction complicating the first rituximab dose the combination therapy with PEG IFN α2a and anti-CD20 monoclonal antibody was well tolerated overall, with no need for IFN dose reduction or discontinuation due to hematological or extra-hematological adverse events. Conversely, a grade 3 cutaneous rash and QT interval prolongation led to early discontinuation of vemurafenib. Vemurafenib-related toxic effects reported in patients receiving vemurafenib both as monotherapy and in association with rituximab include cutaneous rash, photosensitivity, fever, warts, hyperkeratosis, arthralgias or arthritis, fatigue, alopecia, nausea or dyspepsia, and QTc interval prolongation, with most adverse events are mild to moderate in severity. Liver and pancreatic biochemical abnormalities, although frequently reported, are always clinically silent. In a phase 2 study of vemurafenib plus rituximab in patients with relapsed/refractory HCL, the side effects of vemurafenib led to drug dose reduction in almost half of patients, however, most were subsequently able to have the dose re-escalated [8].

Another potential safety issue could derive from the administration of a B cell-depleting agent in the COVID-19 pandemic setting. The patient had a negative history of SARS-CoV-2 infection and had received two doses of the BNT162b2 COVID-19 mRNA vaccine at three-week intervals three months prior to hospital admission. The level of SARS-CoV-2 anti-spike IgG was not assessed. Lymphocytopenia involving B and T CD4+ and CD8+ cell subpopulations might have already existed at the time of vaccination, potentially resulting in impairment of both specific antibody and memory B cell responses and CD4+/CD8+ T cell responses. In the context of PNA contraindication, rituximab in combination with IFN was considered to be most able to induce a rapid hematologic response as a prerequisite for overcoming a life-threatening infection. Moreover, despite B cell depletion secondary to Rituximab, early achievement of full T lymphocyte subset reconstitution is likely to restore antigen-specific T cell responses to SARS-CoV-2 primary infection and vaccination, which may play a protective effect in the absence of humoral response [26].

The clinical and radiological course described in this report is likely to be consistent with IRIS resulting from restored immunity in the setting of an opportunistic infection by *Mycobacterium abscessus.*

IRIS is a common early complication of antiretroviral therapy in HIV/AIDS patients with mycobacterial infections, typically occurring within the first 4–8 weeks of therapy. In the specific setting of NTM infection, IRIS has been described to manifest as fever, lymphadenitis, pulmonary infiltrates, cavitation, and inflammatory masses. Previously-published case definitions for general IRIS and TB-IRIS (tuberculosis-associated IRIS) have been formulated with specific reference to HIV/AIDS patients on ART [4] and as such cannot be applied to non-HIV related conditions, including immunosuppression secondary to hematological malignancies. 

The diagnostic assumption of IRIS in the reported case was based on multiple clinical elements: first, the temporal relationship between the initiation of therapy and the onset of signs and symptoms consistent with NTM-IRIS in the setting of hematological remission and restoration of immune response against the triggering pathogen; and second, the exclusion of alternative diagnoses (antimicrobial drug resistance, recurrent or new infection, drug hypersensitivity reaction) and the prompt response of symptoms to low-intermediate steroid therapy.

Corticosteroids should be considered for treatment of symptomatic IRIS (lymphadenopaty causing airway obstruction, resistant fevers) associated with mycobacterial infections after investigating for other causes for deterioration. A randomized placebo-controlled trial in TB-IRIS demonstrated that the administration of corticosteroids (prednisone 1.5 mg/kg/day for 2 weeks, then 0.75 mg/kg/day for two weeks) reduced the need for hospitalisation and therapeutic procedures and hastened improvements in symptoms, performance, and quality of life [27].

## 4. Conclusions

The efficacy and feasibility of the combination of IFNα-2a in pegylated formulation and rituximab in the setting of frontline treatment of HCL has, at present and to the best of our knowledge, never been reported. The striking rapidity of the hematological response observed makes this chemo-free combination a potentially suitable option in patients with profound immunosuppression and severe infectious complications at onset. The paradoxical effects of prompt immune reconstitution have to be taken into account when dealing with patients with opportunistic infections, and strict surveillance for IRIS during the first several months after immunological recovery is recommended.

Similar to HIV/AIDS patients receiving antiretroviral therapy, patients with severe immunodeficiency secondary to hematologic malignancies and active tuberculosis or NTM infections should generally have specific treatment deferred for at least two weeks after anti-infective therapy in order to limit the adverse effects related to IRIS; this may be particularly important when rapid immune reconstitution is expected as a result of effective immunotherapy strategies.

Further studies on larger samples of patients along with prolonged follow-up are required in order to assess the efficacy of this regimen in terms of both the rates and durability of responses. The recovery of immunocompetence as a requirement for infection control could, in any event, enable patients to receive subsequent treatment with PNAs.

Written informed consent was obtained from the patient for publication of this case report and any accompanying images.
